# Application of Generative Adversarial Networks to Improve COVID-19 Classification on Ultrasound Images

**DOI:** 10.3390/jimaging11120451

**Published:** 2025-12-15

**Authors:** Pedro Sérgio Tôrres Figueiredo Silva, Antonio Mauricio Ferreira Leite Miranda de Sá, Wagner Coelho de Albuquerque Pereira, Leonardo Bonato Felix, José Manoel de Seixas

**Affiliations:** 1 Signal Processing Laboratory, Alberto Luiz Coimbra Institute for Graduate Studies and Research in Engineering/Polytechnic School (Coppe/Poli), Technology Center, Federal University of Rio de Janeiro, Av. Horácio Macedo 2030, Rio de Janeiro 21941-914, Brazil; seixas@lps.ufrj.br; 2 Biomedical Engineering Program, Alberto Luiz Coimbra Institute for Graduate Studies and Research in Engineering (Coppe), Technology Center, Federal University of Rio de Janeiro, Av. Horácio Macedo 2030, Rio de Janeiro 21941-914, Brazil; amflms@peb.ufrj.br (A.M.F.L.M.d.S.); wagner@peb.ufrj.br (W.C.d.A.P.); 3 Department of Electrical Engineering, Federal University of Viçosa, Av. Peter Henry Rolfs s/n, Viçosa 36570-900, Brazil; leobonato@ufv.br

**Keywords:** COVID-19, lung ultrasound, deep learning, generative adversarial networks

## Abstract

COVID-19 screening is crucial for the early diagnosis and treatment of the disease, with lung ultrasound posing as a cost-effective alternative to other imaging techniques. Given the dependency on medical expertise and experience to accurately identify patterns in ultrasound exams, deep learning techniques have been explored for automatically classifying patients’ conditions. However, the limited availability of public medical databases remains a significant obstacle to the development of more advanced models. To address the data scarcity problem, this study proposes a method that leverages generative adversarial networks (GANs) to generate synthetic lung ultrasound images, which are subsequently used to train frame-based classification models. Two types of GANs are considered: Wasserstein GANs (WGAN) and Pix2Pix. Specific tools are used to show that the synthetic data produced present a distribution close to the original data. The classification models trained with synthetic data achieved a peak accuracy of 96.32% ± 4.17%, significantly outperforming the maximum accuracy of 82.69% ± 10.42% obtained when training only with the original data. Furthermore, the best results are comparable to, and in some cases surpass, those reported in recent related studies.

## 1. Introduction

In December 2019, a new form of human coronavirus known as Severe Acute Respiratory Syndrome Coronavirus 2 (SARS-CoV-2) was first detected, which led the World Health Organization (WHO) to declare a global pandemic of the Coronavirus Disease 2019 (COVID-2019) in 2020 [1]. Due to the rapid worldwide spread of the disease, the development of diagnostic tools became urgent, with the reverse transcription polymerase chain reaction (RT-PCR) test adopted as the gold standard for contamination diagnosis [2,3,4]. The gold standard defined by the WHO also recommends serological and radiological testing, with the screening using computerized tomography (CT) and chest X-rays being useful to reveal patterns complementary to the RT-PCR test [5,6,7,8]. Additionally, imaging techniques offer the potential to apply artificial intelligence (AI) methods to improve SARS-CoV-2 detection [3,6,7,8].

Although computed tomography (CT) and chest X-ray are the most commonly used modalities for lung disease screening, their widespread application is limited by challenges such as the requirement for patient mobility and exposure to ionizing radiation [9]. In contrast, lung ultrasound (LUS) does not present these disadvantages, as it enables real-time imaging at a lower cost than CT and X-ray and is portable, making it a good diagnostic tool for regions with limited healthcare systems [10]. The low cost of this kind of exam also allows repeated bedside exams to monitor a patient’s condition and also to help doctors make quick decisions, which is not feasible with CT and X-ray [11]. For these reasons, LUS was also adopted for patient screening during the COVID-19 outbreak, leading to the development of specific protocols to assess disease severity [12]. However, LUS diagnosis strongly depends on the radiologist’s experience and expertise in interpreting visual artifacts [13]. Consequently, this modality has become the focus of studies seeking to enhance the diagnostic process through machine learning approaches.

Some of the earliest studies investigating the use of lung ultrasound (LUS) for COVID-19 diagnosis employed handcrafted feature extraction techniques, focusing primarily on visual patterns such as the pleural line and B-lines [14,15]. These features were subsequently used to train support vector machine (SVM) classifiers, achieving accuracy rates of up to 94% in the work of Carrer et al. [14]. Despite their initial success, such approaches are inherently limited by their reliance on manually defined features, which may introduce bias and information loss by neglecting other potentially relevant image characteristics. Moreover, the dependence on a fixed feature set and static model architecture reduces the adaptability of these methods to patterns or domain changes [16].

Other studies have explored the use of deep learning models that automatically learn image representations directly from data. Almost all of them employed convolutional neural networks (CNNs), which are well known for tasks involving images [3,17,18,19,20,21,22], but there are also approaches that relied on alternatives such as long short-term memory (LSTM) networks [23,24,25,26]. A hybrid framework based on spatial transformers and decision trees was presented in Custode et al. [27]. Regarding the CNN approaches, some used pre-trained models, such as the popular VGG16 explored in Baum et al. [18] and in Born et al. [19], while others employed custom architectures (which was the case for the Mini-COVIDNet presented by Awashi et al. [20] or the XCovNet proposed in Madhu et al. [21]). Although there are similarities regarding the models used, each of these studies introduced important contributions, such as the extensive COVID-19 LUS dataset made available by Born et al. [19] and the development of efficient models for embedded systems in Awashi et al. [20].

While these studies reported promising results and contributed to advance deep learning methods for COVID-19 detection, they were constrained by the scarcity of medical data—a challenge particularly evident in the context of LUS, given the limited number of publicly available datasets (which is explained due to the use of ultrasound as a medical urgency procedure during the pandemic to make quick decisions and usually without preserving the images obtained) [15]. Training deep learning models—which have a massive number of parameters—on datasets with a small number of observations might lead to overfitting, which hinders the use of the model to classify new observation not seen during the training step [28]. One potential way to mitigate this issue is the use of synthetic data generation, with generative adversarial networks (GANs) showing promising results and receiving growing attention in recent years [29,30]. By leveraging adversarial training between two neural networks, GANs are capable of learning rich data representations, thereby alleviating the dependence on large annotated datasets since they can generated synthetic samples based on these data representations that can be used to train the classification models [31]. In the medical domain, recent studies have investigated GAN-based data generation across a variety of imaging modalities, including MRI, CT, and dermoscopy for skin cancer [32,33]. However, there are also applications beyond image synthesis, such as image segmentation, denoising, enhancement, and super-resolution across various examination modalities [33,34].

Several studies have investigated applications of GANs to ultrasound imaging. For data augmentation, these models have been used to generate synthetic images for breast ultrasound, transcranial ultrasound, intraoperative liver ultrasound, and functional ultrasound for neuroimaging, among others [35,36,37,38,39,40]. More recent works have expanded the use of GANs beyond image generation, applying them to a wide range of tasks, including the generation of masks for semantic segmentation of key structures in cardiac ultrasound [41], lesion segmentation in breast ultrasound scans [42], domain adaptation across different ultrasound machines and acquisition protocols [43,44], and speckle noise reduction in ultrasound images [45], while many of these approaches rely on adaptations of established GAN architectures, the most commonly adopted state-of-the-art models include Wasserstein GAN (WGAN), Pix2Pix GAN, CycleGAN, Progressive Growing GAN (ProGAN), and Super-Resolution GAN (SRGAN) [34,46].

Specifically for COVID-19 applications, several works have employed classical GANs and their variants to address the scarcity of imaging data, particularly for X-ray and CT scans [47,48,49,50,51,52,53]. However, only a limited number of studies have reported using GANs to improve COVID-19 diagnosis via ultrasound. In Karar et al. [54], GAN models were trained to improve the COVID-19 classification, using the discriminator to classify the samples and reporting an accuracy of 99.45%. Liang et al. [36] presented a method for generating high-resolution LUS images through the use of information regarding texture and regions of interest for diagnosis. Zhang et al. [55] employed Fuzzy logic as a means to constrain the image generation task, proposing the reference-guided Fuzzy integral GAN (RFI-GAN). Denoising diffusion models were explored in [56] to augment the LUS data, which were used to train a VGG16-based classifier that reached an accuracy of 91%. Fatima et al. [57] proposed an approach named supervised autoencoder generative adversarial networks (SA-GAN), which uses a supervised encoder to build a latent space to minimize the problem of mode collapse (where the network learns the representation of only a small portion of the data distribution) and uses it to augment data for the minority classes, reporting an increase of up to 5% in the classification accuracy for score classification models trained with the synthetic data.

It is important to note that the application of GANs to medical imaging tasks still faces several limitations. Previous studies have highlighted challenges such as training instability in specific GAN variants, long training times, mode collapse, and tendency to overfit the training set [54,58,59,60]. Another concern relates to the evaluation of synthetic data, as commonly used performance measures—such as Fréchet Inception Distance (FID) and Multiscale Structural Similarity (MS-SSIM)—are highly application-dependent and may fail to capture clinically relevant details present in medical images [60]. Finally, a critical limitation that is often overlooked is that, while GANs can synthesize new data consistent with the training distribution and even interpolate across underrepresented regions, there is no guarantee that the synthetic samples will adequately reflect the distributional characteristics of an independent/external dataset, which may contain modes absent from the original training data [61,62].

Delving into the challenge of generating LUS images to improve the COVID-19 diagnosis, this study introduces a novel framework that combines multiple GAN-based synthetic data sources to train a single classifier—a strategy not previously explored in this context. The proposed method integrates two complementary GAN approaches: the Wasserstein GAN (WGAN), known for its robustness against mode collapse [63,64], and the Pix2Pix GAN, which performs image-to-image translation to produce realistic synthetic data [65]. By leveraging the strengths of both models, the framework aims to improve the accuracy and generalization of COVID-19 LUS classifiers. The main contributions of this study are summarized as follows:

- Combining Wasserstein and image-to-image translation GANs to synthesize LUS images.
- Incorporating a new automated extraction of annotated regions from clinically relevant areas of LUS images for Pix2Pix GAN training.
- Proposing a method to verify distributional similarity between generated and real LUS images.
- Evaluating the impact of synthetic data on classifiers’ performance.

Results show a significant performance improvement when a combination of original and synthetic data is used in the training step of the classifiers, achieving results comparable to or even superior to recent studies.

## 2. Materials and Methods

### 2.1. Dataset

The point-of-care ultrasound (POCUS) dataset used in this study comprises LUS exams from 216 patients diagnosed with COVID-19, bacterial pneumonia, viral pneumonia, or healthy/regular conditions [19]. The dataset primarily consists of short videos, although single images represent some exams. Data from viral pneumonia cases and exams acquired with linear transducers were excluded due to their low representation. A summary of the dataset is provided in Table 1.

**Figure 1:**
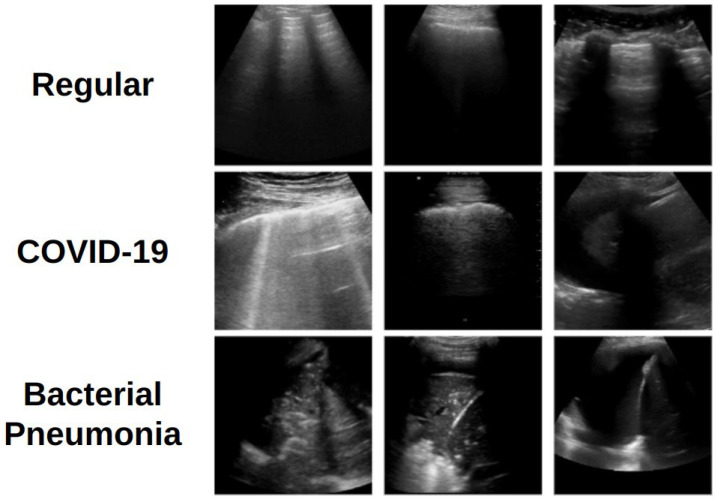
Examples of LUS images representing each of the three classes.

The same preprocessing steps described in [19] have been adopted, with a maximum of 30 frames per video extracted. Each frame was cropped to remove textual annotations and border artifacts and then resized to 128 × 128 pixels to meet computational constraints. Pixel intensities for each image were normalized to the range −1 to +1. A total of 3191 images were extracted (1191 for COVID-19, 708 for bacterial pneumonia, and 1292 for regular/healthy). For training and evaluation, the data were partitioned using stratified 10-fold cross-validation at the patient level, ensuring that no patient appeared in more than one fold. Examples of the preprocessed images are provided in Figure 1.

### 2.2. Generative Models

#### 2.2.1. Wasserstein GAN

The GAN framework proposed by [29] consists of two artificial neural networks (ANNs) trained simultaneously in a zero-sum game. The generator model G(z) is updated to learn the data distribution by mapping a prior distribution $p_{z}\left(z\right)$ (z is typically sampled from a noise process such as white Gaussian noise). The discriminator D(x) is trained to improve its ability to predict the probability that an input x belongs to the real dataset.

Although GANs offer significant advantages over other methods for synthetic data generation, their training can be unstable, heuristic-driven, and prone to mode collapse [63,66]. To mitigate these issues, several alternative formulations have been proposed, such as the WGAN [64], which minimizes the Wasserstein-1 distance, resulting in the optimization process described by Equation (1):

(1) $$W\left(P_{r},P_{g}\right)\approx \min_{G}\;\max_{w\in 𝒲}\;E_{x∼P_{r}}\left[f_{w}\left(x\right)\right]\;-\;E_{z∼p_{z}\left(z\right)}\left[f_{w}\left(G\left(z\right)\right)\right]$$

with $P_{r}$ e $P_{g}$ as the original and generated data distributions, respectively, and 𝒲 being a 1-Lipschitz space [64].

The process described in Equation (1) is only valid when $f_{w}$ satisfies the Lipschitz-1 condition, which was enforced by clipping the weights of the ANN in [64]. However, the clipping constraints limit model capacity and may lead to training instability, as the models end up bound to learn simple functions to satisfy the Lipschitz-1 condition [67,68]. That led to the WGAN Gradient Penalty (WGAN-GP) development, which implemented a new objective function to be minimized during the model’s training [67], presented in Equation (2):

(2) $$L=E_{\tilde{x}∼P_{g}}\left[D\left(\tilde{x}\right)\right]-E_{x∼P_{r}}\left[D\left(x\right)\right]+\alpha E_{\hat{x}∼P_{\hat{x}}}\left[{\left({∥\nabla_{\hat{x}}D\left(\hat{x}\right)∥}_{2}\right)}^{2}\right]$$

with $\hat{x}$ as interpolated observations between the original and synthetic data and α as a penalty coefficient. The third term of Equation (2) adds a penalty necessary for the 1-Lipschitz condition while granting more stability for the WGAN training [67].

**Figure 2:**
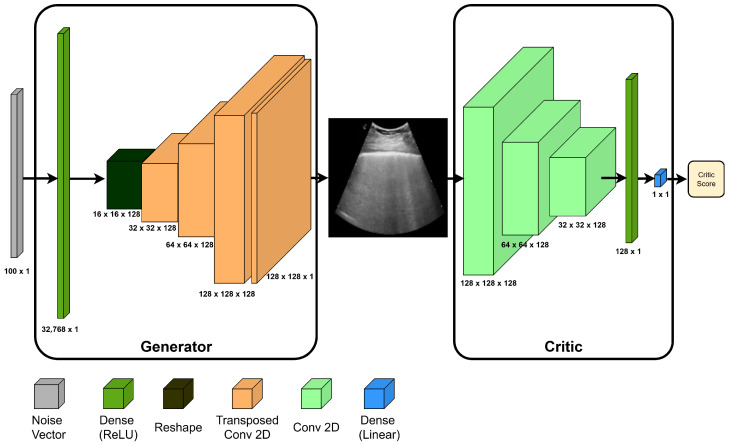
Architecture of the WGAN-GP model implemented.

In the present study, class-specialist WGAN-GPs were trained to enable class-independent data generation. The model topology is shown in Figure 2. The discriminator/critic had three convolutional layers (each with 128 filters, kernels of size 3 × 3, and a stride of 2), one fully connected layer of 128 units, and an output layer composed of a single unit. The rectified linear (ReLU) activation function was used in all layers except the output layer, which had a linear activation function. For the generator network, there was a fully connected layer with 32,768 units, followed by three transpose convolution layers (128 filters, 3 × 3 kernel, and stride of 2) and the output was produced by a final transpose convolutional layer (1 filter, 3 × 3 kernel size, and stride of 1). The hyperbolic tangent activation function was used in the output layer, while ReLU was employed in all the other layers. The generator received a 100-dimensional vector as input, sampled from a spherical Gaussian distribution. Batch normalization was applied exclusively to the generator’s layers.

Each model was trained using a batch of 64 images and the Adam optimizer (learning rate = 0.0001, β1 = 0.5, β2 = 0.999). During training, the critic was updated five times for each update of the generator. Once training was completed, the generator was employed to produce synthetic data.

#### 2.2.2. Pix2Pix GAN

Pix2Pix GAN, or shortly Pix2Pix, was presented by Isola et al. [65] as an adaptation of the GAN framework for image-to-image translation tasks. It builds upon the conditional GAN (cGAN), in which the generator is trained to map the observed data x and random noise z onto the output image y (G:x,z→y) [69]. The objective function for *cGAN* is given in Equation (3):

(3) $$L_{cGAN}\left(G,D\right)=E_{y}\left[logD\left(y\right)\right]+E_{x,z}\left[log\left(1-D\left(G\left(x,z\right)\right)\right)\right].$$

Pix2Pix introduces some modifications to the *cGAN* framework. Notably, the noise term z is incorporated only through dropout within the model instead of being an explicit input. Another change is the addition of the *L*1 term to the equation above, which results in the objective of Equation (4):

(4) $$L_{Pix2Pix}=arg\underset{G}{min}\;\underset{D}{max}\;L_{cGAN}\left(G,D\right)+\lambda L_{L1}\left(G\right)$$

with λ as a penalty coefficient and $L_{L1}$ as the *L*1 distance between the generated and original data.

**Figure 3:**
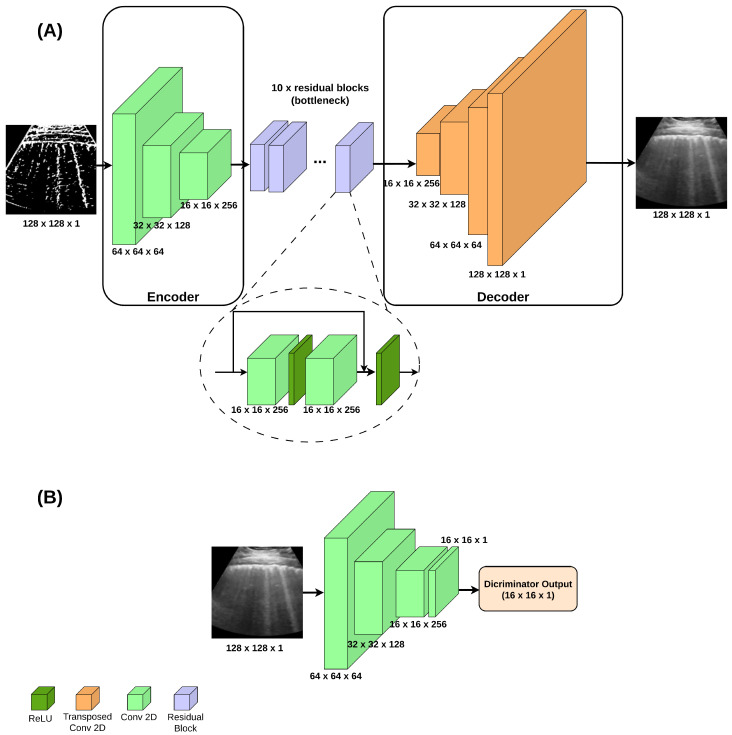
Generator (**A**) and discriminator (**B**) topologies for Pix2Pix model.

The Pix2Pix generator adopts an encoder-decoder structure. As for the discriminator, it operates at the patch level (PatchGAN, as proposed in the original paper), averaging local classifications to compose the final output. A generator architecture inspired by [36] was adopted in this study, which employed residual blocks at the bottleneck of the encoder-decoder. The topologies for the Pix2Pix generator and discriminator are shown in Figure 3. Batch normalization and dropout (0.5) were included after each convolutional layer, which used a 3 × 3 kernel. The ReLU activation function was applied on each convolutional layer.

#### 2.2.3. Image Processing for Pix2Pix

**Figure 4:**
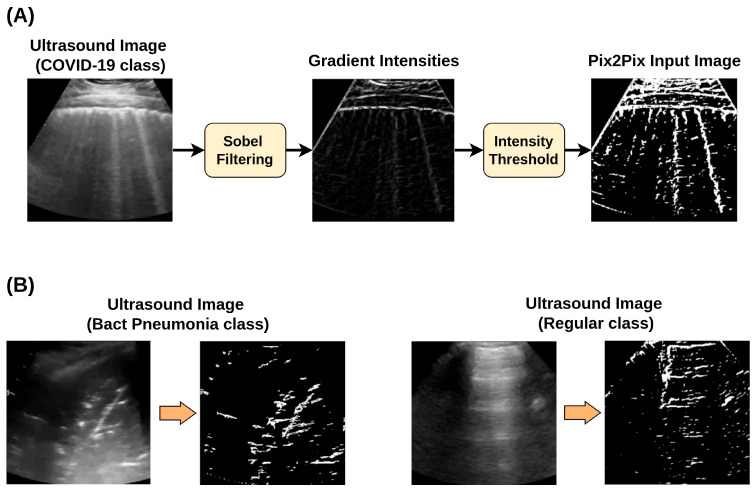
(**A**) Sobel filtering + threshold processing pipeline and (**B**) examples obtained when applying to the other two classes.

Pix2Pix performs image-to-image translation and requires an input image to produce a corresponding output. In the context of medical data generation, the model can use annotated regions of interest as inputs [36]. Unfortunately, such annotations were absent from the dataset used in this study, which prompted the authors to devise a method to generate annotations for LUS artifacts. This was achieved by applying Sobel filtering to the image, which highlights its intensity gradients. As the main diagnostic LUS artifacts are hyperechoic, this filtering effectively highlights them. The resultant image was converted to a binary image using a threshold at the 90th percentile of pixel intensity (pixels in the 90th percentile were set to 1, while the remaining pixels were set to 0). An illustration of this processing method, along with some examples of its results, is shown in Figure 4.

**Figure 5:**
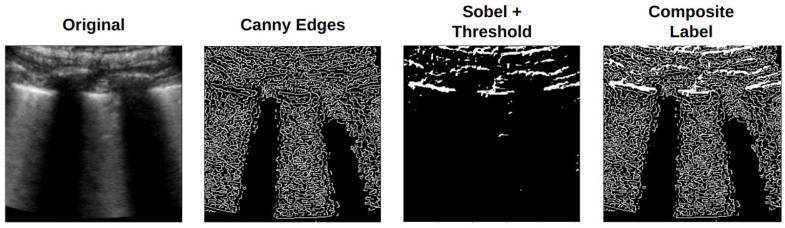
Original LUS image and respective Pix2Pix input labels obtained for each processing method.

Although the described processing aimed to capture the location of specific LUS artifacts, it may have discarded fine-grain texture information that could also be clinically relevant [36]. To account for this, the Canny method for edge detection used in Liang et al. [36] was also employed. Accordingly, three different scenarios for Pix2Pix training were evaluated: (i) using images produced by Sobel filtering followed by pixel thresholding (Sobel + threshold), (ii) images derived exclusively from Canny edge detection (Canny edges), and (iii) a combined approach integrating both methods, referred to as composite labeling. Examples are shown in Figure 5.

### 2.3. Classification Models

To evaluate whether the generated data could enhance the performance of COVID-19 LUS classifiers, two training approaches were tested: one using only the original LUS data, and another combining synthetic and original data. WGAN and Pix2Pix data were initially used separately and later combined. The study employed the VGG16-based pretrained model described in [19] and the XCovNet architecture introduced in [21] as classifiers.

The VGG16-based classifier is essentially a CNN pre-trained in the Imagenet dataset [70,71]. New fully connected layers were added to the model: a hidden layer with 64 neurons using the ReLU activation function and an output layer with three units and a softmax activation function. For the XCovNet model, the same topology described in [21] was used, with only the input size adjusted for 128 × 128 images. Full descriptions of the classifiers’ architectures are shown in Table 2. Batch normalization and dropout regularization were used during the training. Both models were trained for 40 epochs using the categorical cross-entropy objective function, Adam optimizer (learning rate = 0.0001), and a batch size of 16 images. Early stopping was enabled using a validation set.

When training the classifiers, standard affine transformations were applied to balance the classes and improve generalization [57]. These transformations included random translation (up to 15%), rotation (up to 15°), horizontal flipping, and scaling (up to 45%), applied only to copies of the original samples in the training set. Additionally, since the classifiers’ architectures required three-channel inputs, each grayscale image was replicated across all three channels to match the expected input format.

### 2.4. Cross-Validation and Performance Measures

**Figure 6:**
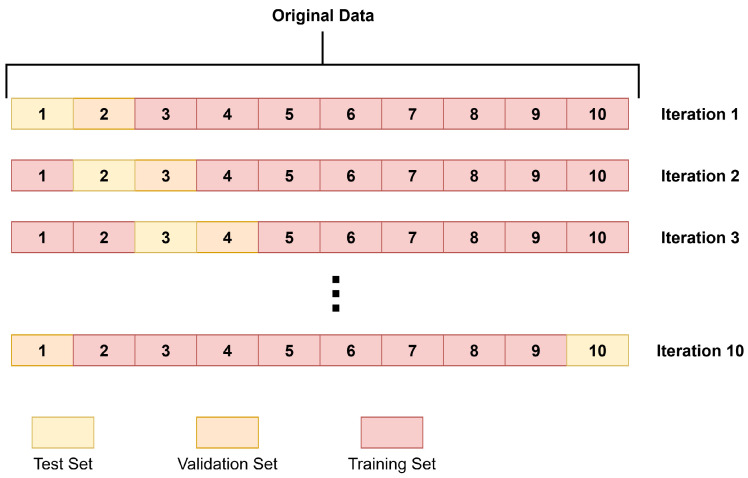
K-fold cross-validation splits on each iteration.

**Figure 7:**
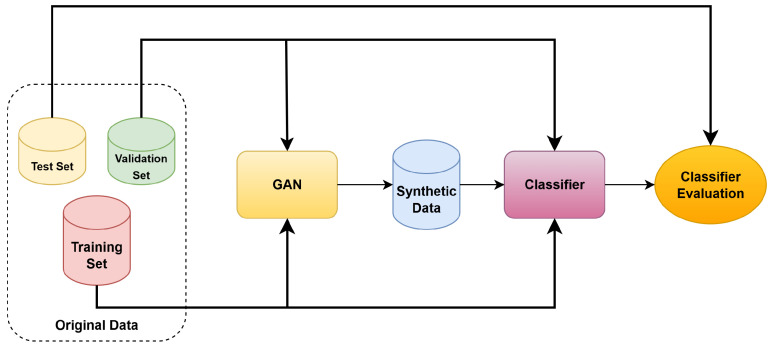
Training process for one k-fold cross-validation iteration.

As previously mentioned, a patient-level stratified k-fold cross-validation (k = 10) has been used to split the data for training both the GAN and classifier models. The experimental data were divided into k disjoint subsets with a similar number of patients. In each iteration, one of these subsets was designated as a test set (hold-out), another as the validation set (used for hyperparameter tuning and early stopping), and the remaining subsets as the training set. Figure 6 illustrates the k-fold data split, while Figure 7 depicts the whole training process repeated across all iterations. As shown in Figure 7, both the GAN and the classifier used the same training, validation, and test partitions, preventing data leakage when training the classifier with synthetic data.

Classifier performance was evaluated using accuracy, sensitivity, and specificity, which are the most commonly reported measures in similar studies [9,15]. Since it is also essential to check whether or not the models are focusing on clinically known artifacts for the classification task, Gradient-weighted Class Activation Mapping (Grad-CAM) was used to evaluate image regions that caused class activity [19,72]. However, there remains considerable debate on how to properly measure the performance of generative models in terms of the variety and quality of the generated data [73]. The present work adopted an evaluation method inspired by [74], employing the Kullback–Leiber (KL) divergence along with the L1 and L2 norms.

**Figure 8:**
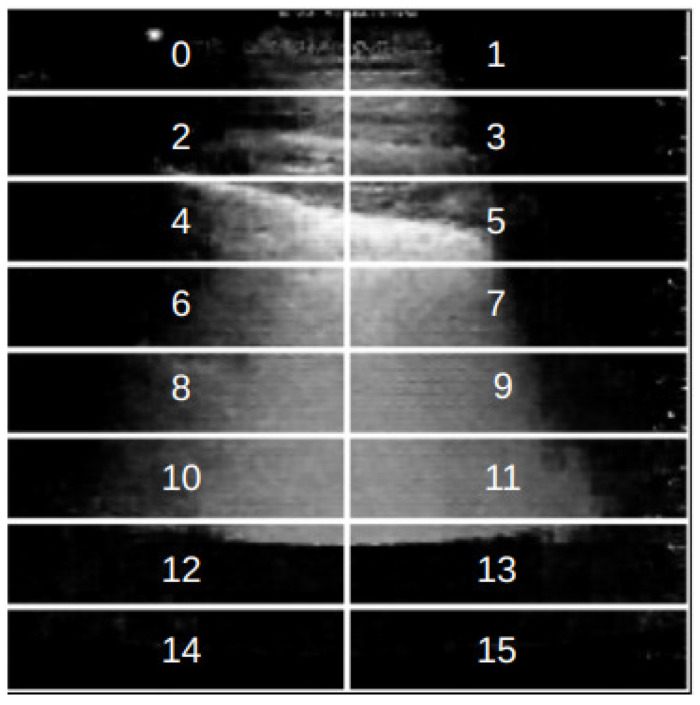
Example of a LUS image divided into the defined indexed patches.

For the generated images, it is desired to measure if they follow the same probability distribution function as the original data. To that purpose, the *KL* divergence served as a similarity measure between any pair of images, as described by Equation (5):

(5) $$D_{KL}\left(P||Q\right)=\sum_{x\in x}P\left(x\right)\;log\left(\frac{P(x)}{Q(x)}\right)$$

with *P* and *Q* as two probability distributions defined in the sample space X. The main idea is to verify whether the fluctuations of the *KL* divergence estimated from the synthetic data fall within the fluctuations estimated from the original data. To do that, the following procedure was devised:

- each image was divided into non-overlapping patches of size 56 × 14 pixels, as shown in Figure 8;
- for each pair of original images in the training set, the KL divergence was computed at the patch level, comparing the distribution of pixels for the two images in corresponding patches;
- the same procedure was repeated for each original-synthetic pair and each synthetic-synthetic pair, always using the original images contained in the training set;
- The three sets of the KL divergence values obtained for each patch were used to check whether the synthetic data fluctuations are close to those estimated from the original data.

Given the importance of LUS artifacts for diagnosis (such as the pleural line and B-lines), the images were divided into patches rather than comparing the full images using Equation (5). The patch size used was such that the pleural line was contained in one or two consecutive patches, resulting in 16 patches per image. The KL divergence computation estimates whether the fluctuations for patch regions of the synthetic data are within the fluctuations from the original dataset. If so, it would indicate that the generated data followed the probability density function (PDF) of the original data for such regions.

While the KL divergence can serve as a similarity measure between images, it does not indicate whether the generator merely replicated the images from the training set. If that were to happen, the variance estimated from original-synthetic and synthetic-synthetic pairs would be the same as that estimated from original image pairs. To verify this, the L1 and L2 norms were calculated pixel-wise for each pair of original-original and original-synthetic images. Thus, if the generator only reproduces samples from the training set, the L1 and L2 norms for original-synthetic pairs would be very close to zero.

The evaluation was further complemented by the Kernel Inception Distance (KID), which is similar to the popular Fréchet Inception Distance (FID) but is unbiased for small datasets [36,75]. KID is computed by comparing two sets of images after passing them through an Inception-v3 network, excluding its top layers [75]. Lower KID values indicate greater similarity between the distributions of the compared image sets. In this study, KID was calculated between the generated images and the test set for each k-fold iteration. Additionally, a baseline KID was obtained by comparing the training and test sets within each cross-validation fold.

## 3. Results

### 3.1. Synthetic Data Generation

#### 3.1.1. WGAN

**Figure 9:**
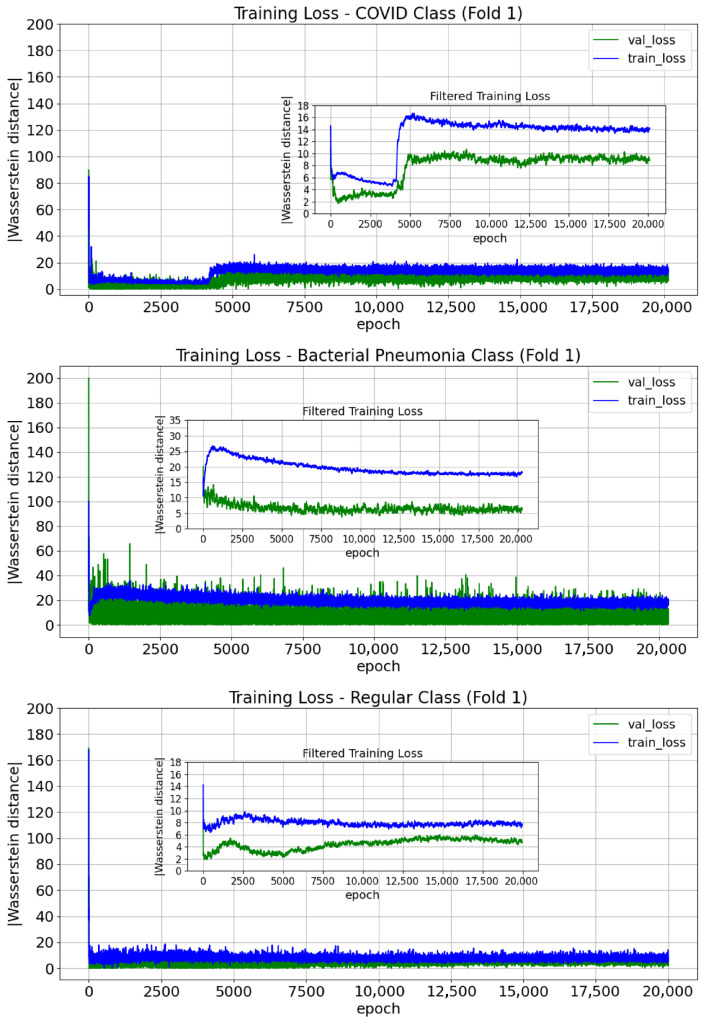
Training curves for the class expert WGAN-GPs trained in the first iteration of the k-fold cross-validation.

The WGAN training curves for a representative k-fold iteration are shown in Figure 9. To better assess loss convergence, a moving-average filtered version (50-epoch window) is also presented, indicating successful training [63,67,74]. Although differences are observed across the three plots—particularly during the initial epochs—a common pattern emerges: a rapid decay at the start, followed by an increase in training loss, and then a gradual decline towards the end of training. Since the estimated Wasserstein distances do not reach zero, differences persist between the original and synthetic PDFs, which may benefit classification by introducing additional diversity to the training data when synthetic images are used [74].

**Figure 10:**
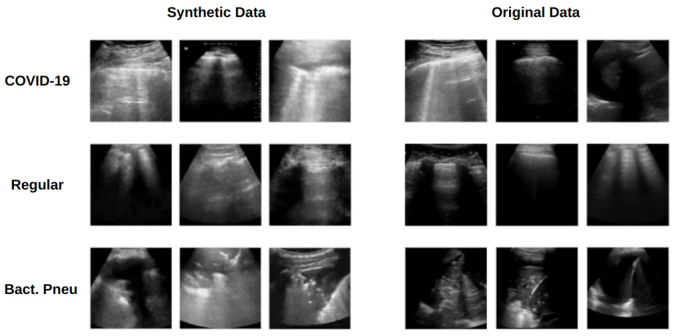
Comparison of original and synthetic ultrasound images generated using the WGAN-GP models.

**Figure 11:**
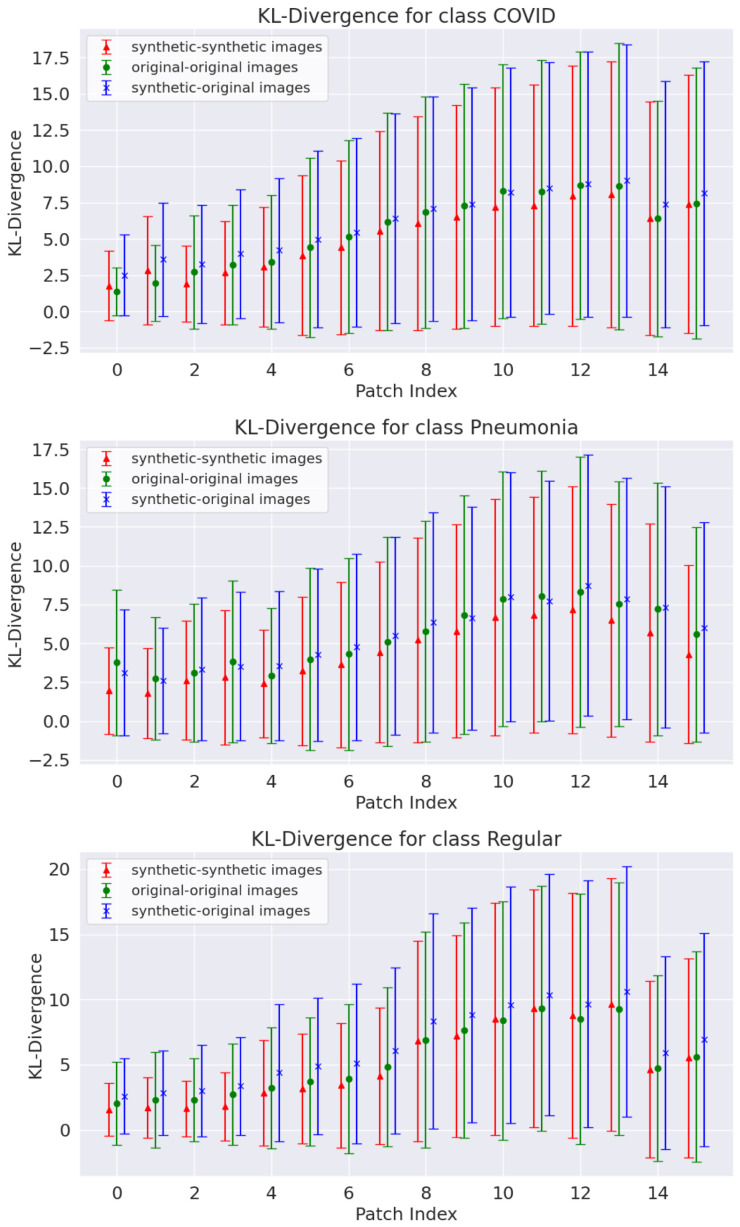
Patch-level KL divergence for pairs of original and synthetic images for the three classes (results for fold 1 of k-fold cross-validation).

The trained generators produced 5000 images per class in each k-fold iteration (for comparison, the original dataset contained 3191 images across the three classes). Some synthetic examples are compared to the original images in Figure 10. The synthetic images resemble the original data, replicating LUS artifacts commonly used in medical diagnosis, although many generated images still lack B-lines—observations that some LUS specialists validated. This is particularly important since the LUS diagnostic protocols rely on the presence and quantification of these artifacts [12]. Figure 11 shows a patchwise comparison of the KL divergence fluctuations for all pairs of original, synthetic, and original-synthetic images. The graphs show that the fluctuations of synthetic data are similar to those of original data across patches.

**Figure 12:**
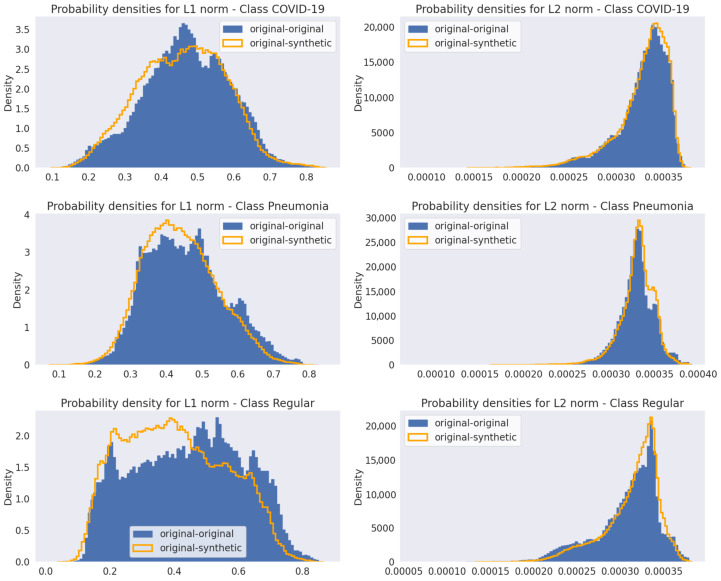
L1 and L2 norms probability densities for original-original and original-synthetic images for the first fold of the k-fold cross-validation.

Figure 12 presents the probability densities estimated for L1 and L2 norms in the first fold of the cross-validation. It is noticeable that the distributions of the differences between original and synthetic data closely resemble those of the original data, with only minor variations.

#### 3.1.2. Pix2Pix

**Figure 13:**
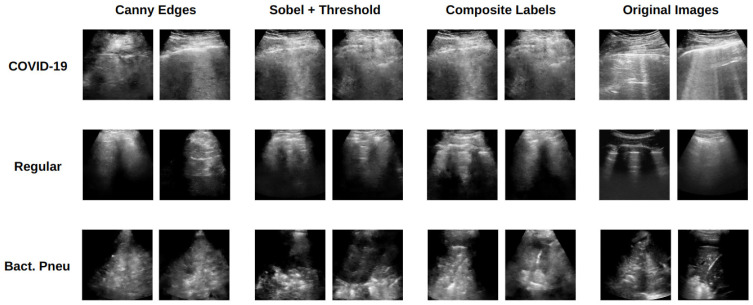
Pix2Pix U-net generated images for the three processed inputs used. Images from the training set are displayed in the last column for comparison.

Since Pix2Pix models lack an interpretable measure like the Wasserstein distance used in training, they were initially trained for 2000 epochs. The trained generators were employed to produce a new image for each image in the training set, and the same evaluation applied to the WGAN models was performed for the Pix2Pix-generated data. Figure 13 presents examples of generated images for each processed map and across the three classes. The synthetic images are similar to the original ultrasound but lack B-lines (which were points highlighted by some medical specialists). This could be caused by the B-lines being less echogenic than the pleural lines, A-lines, and consolidation spots [76].

**Figure 14:**
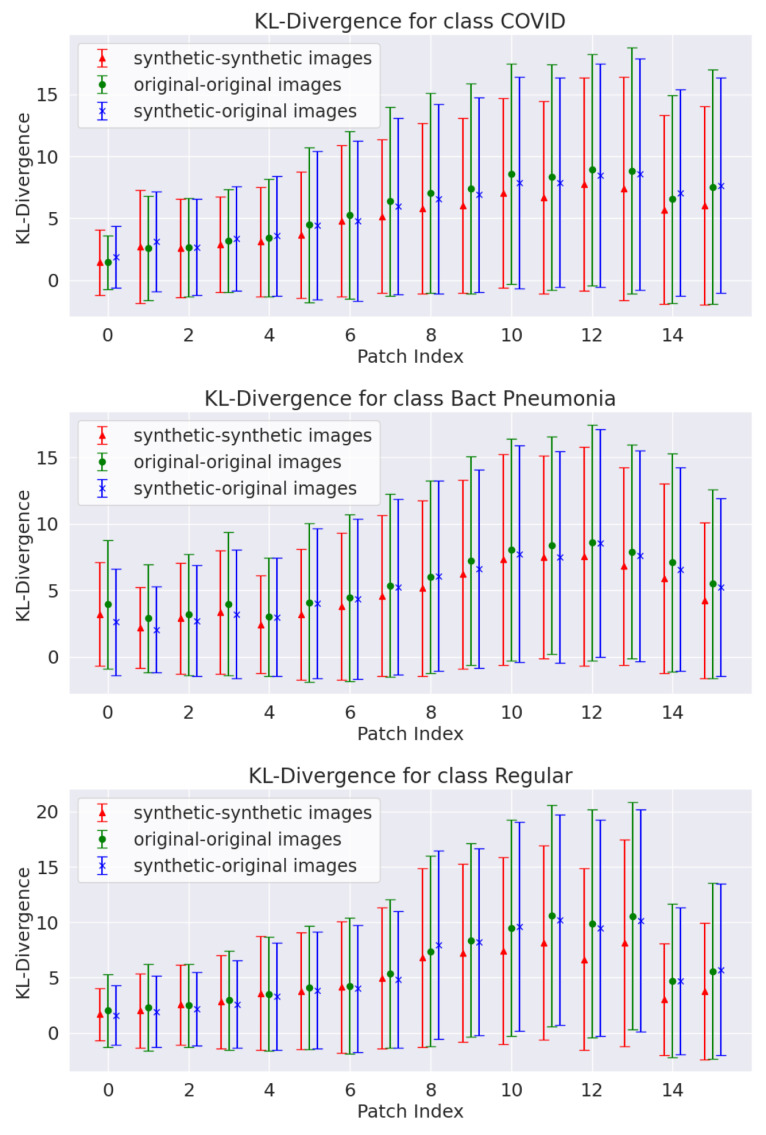
Patch-level KL divergence for pairs of original and synthetic images generated by the Pix2Pix model using the Canny edges input maps (results for fold 1 of k-fold cross-validation).

The same KL divergence and L1 and L2 norm analyses performed for WGAN-generated data were repeated for the Pix2Pix data. Figure 14 shows the KL divergence results for the Canny edge input maps, with the other two input maps showing similar behavior. As seen with the WGAN models, the patchwise KL divergence variability is similar to that estimated for the original data. This result was consistent across the three different Pix2Pix inputs and for each class present in the original data.

**Figure 15:**
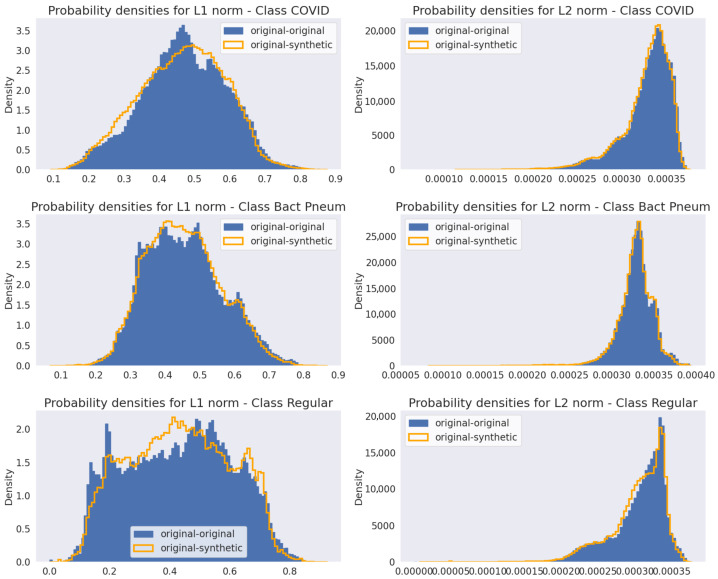
L1 and L2 norms probability densities for original-original and original-synthetic images using the Pix2Pix models with Canny edges input maps (results for the first fold of the k-fold cross-validation).

The L1 and L2 norm analyses for the Pix2Pix models using Canny edge input maps are shown in Figure 15, with the other input maps showing similar results. As observed for the WGAN models in Figure 11, the probability densities of the norms comparing original-synthetic image pairs are very close to those from the original data, with only minor differences.

#### 3.1.3. KID Evaluation

Table 3 reports the average KID values and standard deviations across folds, along with a baseline derived exclusively from the original data. For the COVID-19 class, the WGAN-GP method achieved the lowest mean and standard deviation, while the Pix2Pix-based approaches showed comparable averages and error margins. For the bacterial pneumonia and healthy classes, WGAN-GP and the Composite Labels method yielded similar KID scores. Statistical analysis using the Wilcoxon signed-rank test indicated significant differences between WGAN and the other methods in most cases, except in comparisons with Composite Labels for bacterial pneumonia and healthy classes (*p* < 0.05). Within the Pix2Pix group, the Composite Labels method differed significantly from the other Pix2Pix variants for both the bacterial pneumonia and healthy classes. Moreover, the baseline was statistically different from all approaches for these two classes. However, for the COVID-19 class, a significant difference was observed only between the baseline and the WGAN method.

### 3.2. Classification Performance

Table 4 presents results comparing scenarios in which models were trained on the original dataset alone and on the original dataset plus WGAN-GP–generated synthetic data. The inclusion of synthetic images resulted in a notable reduction in variance and a consistent improvement in average classification performance across all evaluation metrics. The XCovNet classifier exhibited relatively low performance when trained solely on the original data, in contrast to the results reported by Madhu et al. [21] using the same dataset. This discrepancy can be attributed to methodological differences: the present study employed k-fold cross-validation at the patient level, ensuring that samples from a single patient did not appear in both training and test sets—an approach not adopted in [21], which may have introduced data leakage across partitions. When the same classifier was trained without patient-level partitioning, performance levels aligned closely with those reported in [21] (Accuracy: 99.06% ± 0.4%, COVID-19 Sensitivity: 0.99 ± 0.01, COVID-19 Specificity: 0.99 ± 0.02). Finally, a Wilcoxon signed-rank test confirmed that the inclusion of synthetic data yielded statistically significant improvements across all performance measures, rejecting the null hypothesis at a 95% confidence level.

The results for the classification models trained using Pix2Pix synthetic data are shown in Table 5. Again, a clear performance improvement is observed when incorporating synthetic data, compared to the baseline classifiers trained solely on the original dataset, as presented in Table 5. However, the results obtained for the three input map variations in the Pix2Pix model showed minimal differences. When comparing the results two by two using the Wilcoxon signed-rank test, it was impossible to reject the null hypothesis when comparing the values obtained using the Canny edges and Sobel edges input maps (95% confidence level). However, the same is not true when comparing the last two with the results of the composite label input maps. Furthermore, the null hypothesis for the same test can be rejected when comparing any classifier trained on Pix2Pix data to the classifier trained only on original data.

The Wilcoxon signed-rank test was also applied to compare each result obtained with Pix2Pix to those from WGAN-GP. Surprisingly, the null hypothesis could not be rejected in any of the three comparisons, which makes it impossible to say that any of the Pix2Pix results were better than the WGAN result.

A final scenario is shown in Table 6, where the classifier model is trained using the original data and synthetic data produced by WGAN and Pix2Pix generators. There is an apparent general improvement compared to the training using WGAN or Pix2Pix data individually. However, the Wilcoxon signed-rank test did not point out a significant difference when comparing the results in Table 6 to the values in Table 4 and Table 5.

In Table 7, there is a comparison with recent studies that addressed similar classification tasks. The approaches using synthetic data presented in this paper achieved performance comparable, if not better than, some of these other works. It is important to note that the classifier used in this study was the same as that used in [19] for the image classification task. Furthermore, the results of Born et al. [19] and Bruno et al. [77] employed cross-validation at video level, which may allow data of a given patient to be present in more than one fold.

**Figure 16:**
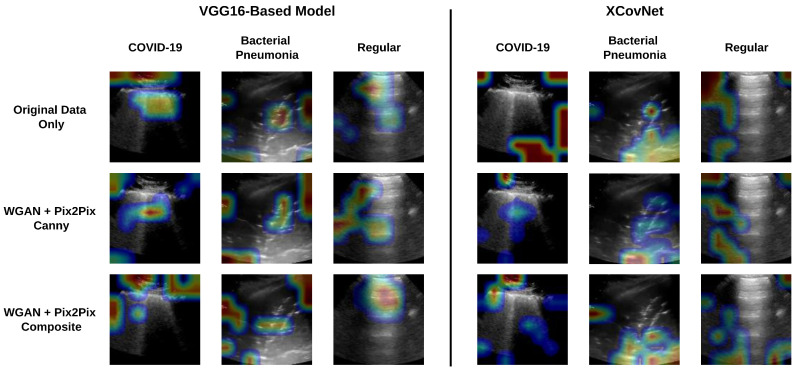
Examples of Grad-CAM regions of interest for each class and each classifier.

Finally, Grad-CAM was employed for model explainability for the trained classifiers. As shown in Figure 16, VGG16-based classifiers appear to focus more on the medical artifacts of interest (pleural line, A-lines, B-lines, and consolidation spots) even when trained solely on the original data. On the other hand, it is possible to observe improvements in the regions of interest for XCovNet when using synthetic data, particularly for the COVID-19 class. Table 8 shows the averages and dispersions for the activation maps’ centroids of all images in the dataset. In general, the classifiers present a centroid within the patches 4 and 6 in Figure 8, which is generaly where the pleural line is contained.

## 4. Discussion

Much of the Results Section is devoted to evaluating the generated data, as the best approach for assessing generative models remains an open topic. The present study adopted the KL divergence to assess data diversity in a distribution. The quasi-distance was measured between image patch pairs, and estimates for synthetic data and original–synthetic pairs were found to be similar to those observed in the original data. This suggests that the synthetic data produce fluctuations similar to those from original data within each analyzed region. Furthermore, the L1 and L2 norm analyses confirm the absence of replicas of the training data (as no zero entries were observed in any histogram) and demonstrate that the distributions derived from original data are close to those obtained from synthetic data.

The KID analysis revealed that, although the images generated by the Pix2Pix approaches were generally closer to the original data distribution, the WGAN method demonstrated a notable advantage. Interestingly, the average KID for the WGAN-generated data was lower than the KID estimated in the original dataset. This suggests that the distribution of the WGAN-generated images may be closer to the original data than the distance observed between different subsets of the original data, while this could reflect limitations of the KID metric—such as its reliance on a model pretrained on the broad ImageNet dataset and the required resizing of images to fit the Inception V3 input, which may affect image fidelity—it may also indicate that WGAN-GP is capable of generating samples that fill distributional gaps, particularly those arising from partitioning data across a limited and heterogeneous patient population.

As demonstrated in the Results section, the WGAN and Pix2Pix models provide compelling evidence of their potential to mitigate the scarcity of medical imaging data. The classifier architectures and hyperparameters were kept consistent with those used in [19,21], yet a notable performance improvement was achieved when training with a combination of original and synthetic data, as presented in Table 4, Table 5 and Table 6. This enhancement can be attributed to the data augmentation effect introduced by the synthetic samples, which increases the number of training observations and thereby reduces the risk of overfitting during model training. Moreover, because WGAN and Pix2Pix models are trained to approximate the underlying probability distribution of the real data, the generated images are not simple transformations of existing samples but instead introduce novel, distribution-consistent variations. This contributes to greater data diversity, thereby enhancing model generalization on unseen test data.

Although Table 7 shows that some studies may outperform the results of the method presented in this paper, it is important to remember that the cross-validation used was very rigorous, partitioning the data at the patient level and not allowing test data to leak at any point for the training of the models. Unfortunately, that extra care seems not to be taken in related studies, such as Karar et al. [54], Madhu et al. [21]. Furthermore, the possibility of generating new data is a powerful tool that can benefit even the models with the highest performance to date.

The comparison with similar studies is limited by the reduced number of studies and the diversity of performance measures adopted [36,54,56,57]. Thus, a comparison can be made with Karar et al. [54] and Zhang et al. [56], which used GANs to generate new data to improve the training of COVID-19 classifiers. As shown in Table 3, this study’s method yields average results above those of Zhang et al. [56], although both studies overlap in their error bars. Karar et al. [54], reported some of the best results for the classification task, but since a k-fold at patient or video-level was not adopted, the values for the performance measures might be optimistic. Even so, the method presented in this paper still reached results close to those of Karar et al. [54], when considering the error bar.

The WGAN and Pix2Pix approaches yielded similar results, but some significant differences were observed. First, since WGAN generators map random noise onto a LUS image, a large number of observations can be generated. This is not the case with Pix2Pix, as its trained models require an input map to generate an output LUS image. Another difference between these models emerged during training: WGAN models required significantly more epochs to generate realistic LUS images, while Pix2Pix models, after just 2000 epochs, already produced data that enhanced classifier performance. Thus, there is a trade-off between fast training and the number of generated samples.

In particular, for Pix2Pix models, it was surprising that the results for composite label input maps were slightly lower on average compared to the other two preprocessing techniques. When checking for significant differences, the null hypothesis of the median difference between paired observations being equal to zero could be rejected when comparing the composite label results to the other two, meaning the composite label results can be lower than the other Pix2Pix results. This was unexpected, as this approach integrates information from the other two input maps. However, the overlap of input maps could hide information regarding the localization and shape of the artifacts of interest. This is an interesting point that will be addressed in future studies.

The activation maps analysis has shown that, in general, the trained classifier models tend to focus on the center of the input images, which aligns with the position of the pleural line, B-lines, and A-lines. However, as shown in Figure 16, other regions of the images also generate strong activation. Particularly for the XCovNet trained only with the original data, the activation map shown highlights only regions in the corners of the image, which do not present any meaningful artifacts. However, the same model seems to focus more on regions of the clinical artifacts when trained with the synthetic data.

Training the classifiers with both WGAN and Pix2Pix data resulted in a slight improvement in the average estimates from k-fold cross-validation. This suggests that synthetic data from both sources may complement each other, covering modes that each alone misses. However, further investigation is needed, as the Wilcoxon signed-rank test showed no significant difference between the combined approach and the individual use of WGAN or Pix2Pix synthetic images.

There are some limitations that should be acknowledged. First, there is still a high cost of computer resources and training time, which were faced when training the GAN models. This limited the image resolution and, particularly for the WGANs, increased the time to train the model by weeks—models were trained on NVIDIA RTX 3070 GPUs. Second, although the quantitative analyses indicate that the distributions of synthetic and real images are comparable, visual inspection reveals that some generated samples lack characteristic medical artifacts, such as well-defined B-lines. This suggests a risk of artifact distortion or omission, reflecting an intrinsic limitation of the employed GAN architectures in accurately replicating subtle diagnostic features. Third, the restricted dataset size imposes constraints on the representativeness of the training data, potentially limiting the model’s ability to capture the full variability of lung ultrasound (LUS) patterns across different populations, acquisition settings, and imaging devices. This limitation may, in turn, affect the generalizability of the trained models to broader clinical contexts. Nonetheless, as noted by Born et al. [19], the dataset used in this study presents a certain degree of heterogeneity in terms of patient metadata, technical parameters, and disease progression, which partially mitigates the limitations associated with dataset size and homogeneity.

## 5. Conclusions

Deep learning techniques hold significant potential for enhancing the screening and diagnosis of COVID-19 through lung ultrasound (LUS) analysis. However, their performance remains constrained by the limited availability of publicly accessible medical imaging datasets. To address this challenge, the present study introduced a novel framework that integrates two complementary GAN-based generative models to produce synthetic LUS images, thereby augmenting the training data available for deep learning classification and improving overall model performance. In addition, the study proposed quantitative measures to assess the distributional similarity between original and synthetic images, providing a foundation for evaluating the fidelity and representativeness of the generated data.

WGAN and Pix2Pix models were trained to generate synthetic data. An automated preprocessing pipeline was developed to extract annotated regions corresponding to clinically relevant LUS artifacts, which were subsequently used as input for the Pix2Pix model. The generated images demonstrated a high degree of visual and statistical similarity to the original data, presenting comparable variance and distribution characteristics. When these synthetic images were combined with real data for classifier training, the resulting models achieved significant performance gains over baselines trained exclusively on the original data. Moreover, the proposed approach yielded results comparable to, and in some cases surpassing, the best-performing methods reported in recent literature, underscoring its effectiveness and practical potential in medical image analysis.

Although certain limitations persist regarding the computational cost of training these models and the relatively small dataset size, the proposed approach demonstrates potential to enhance the performance of classifiers developed for this task, while lung ultrasound (LUS) is already a low-cost alternative to CT and X-ray imaging, using synthetic data can further reduce reliance on large volumes of patient data for training classification models. This, in turn, can support the development of computer-aided diagnostic (CAD) systems that may be integrated into portable devices, enabling deployment in regions with limited access to healthcare technologies.

Future work will aim to overcome the current methodological limitations regarding the representation of B-lines in synthetic images. This will involve using manually annotated regions corresponding to these artifacts as auxiliary information during the training of generative models, enabling the exploration of architectures such as the Auxiliary Classifier GAN (ACGAN) and the Conditional GAN (CGAN). Additionally, the authors plan to extend the proposed framework to generate short synthetic LUS videos by incorporating 3D convolutional neural networks (3D CNNs) and experimenting with VideoGAN architectures. This extension is expected to capture the temporal dynamics inherent to LUS examinations, which are critical for comprehensive clinical assessment and diagnostic accuracy.

## Figures and Tables

**Table 1:** Composition of the LUS Dataset Used. Adapted from [19].

	Convex Transducer		Linear Transducer		
Class	Video	Image	Video	Image	Sum
COVID-19	64	18	6	4	92
Bact. Pneu.	49	20	2	2	73
Viral Pneu.	3	-	3	-	6
Healthy	66	15	9	-	90
Total	182	53	20	6	261

**Table 2:** Layer-by-layer architecture comparison between VGG16 [71] and XCovNet [21].

Stage	VGG16 Layers	XCovNet Layers
Input	Input 128×128×3	Input 128×128×3
Block 1	Conv2D (64, 3×3) + ReLU Conv2D (64, 3×3) + ReLU MaxPool (2 × 2)	Conv2D (32, 3×3) + PReLU Conv2D (32, 3×3) + PReLU MaxPool (2 × 2)
Block 2	Conv2D (128, 3×3) + ReLU Conv2D (128, 3×3) + ReLU MaxPool (2 × 2)	SeparableConv2D (64, depthwise 3×3 + pointwise 1×1) + PReLU SeparableConv2D (64) + PReLU MaxPool (2 × 2)
Block 3	Conv2D (256, 3×3) + ReLU Conv2D (256, 3×3) + ReLU Conv2D (256, 3×3) + ReLU MaxPool (2 × 2)	SeparableConv2D (128) + PReLU SeparableConv2D (128) + PReLU SeparableConv2D (128) + PReLU MaxPool (2 × 2)
Block 4	Conv2D (512, 3×3) + ReLU Conv2D (512, 3×3) + ReLU Conv2D (512, 3×3) + ReLU MaxPool (2 × 2)	SeparableConv2D (256) + PReLU SeparableConv2D (256) + PReLU SeparableConv2D (256) + PReLU MaxPool (2 × 2)
Block 5	Conv2D (512, 3×3) + ReLU Conv2D (512, 3×3) + ReLU Conv2D (512, 3×3) + ReLU MaxPool (2 × 2)	SeparableConv2D (512) + PReLU SeparableConv2D (512) + PReLU SeparableConv2D (512) + PReLU MaxPool (2 × 2)
Classification Head	Flatten Dense (64) + ReLU Dense (3) + Softmax	Flatten Dense (512) + PReLU Dense (128) + PReLU Dense (64) + PReLU Dense (3) + Softmax

**Table 3:** Evaluation of generated data using KID.

Method	COVID-19	Bact. Pneu.	Healthy
WGAN-GP	0.794 ± 0.240	0.555 ± 0.204	0.336 ± 0.153
Pix2Pix Canny Edges	1.394 ± 0.485	0.790 ± 0.294	0.494 ± 0.216
Pix2Pix Sobel + Threshold	1.440 ± 0.484	0.842 ± 0.272	0.492 ± 0.189
Pix2Pix Composite Labels	1.221 ± 0.614	0.617 ± 0.241	0.354 ± 0.229
Baseline (Original Data)	1.203 ± 0.820	0.447 ± 0.253	0.218 ± 0.082

**Table 4:** Results for the classifier models trained with original data only and a mix of original and WGAN synthetic data.

**Pretrained VGG-16 Classifier**				
	**Original Data Only**		**WGAN + Original Data**	
**Accuracy**	82.69% ± 10.42%		91.65% ± 4.05%	
	**Sensitivity**	**Specificity**	**Sensitivity**	**Specificity**
COVID-19	0.81 ± 0.10	0.87 ± 0.13	0.91 ± 0.11	0.95 ± 0.06
Bact. Pneu.	0.78 ± 0.13	0.92 ± 0.04	0.92 ± 0.06	0.97 ± 0.04
Healthy	0.78 ± 0.21	0.87 ± 0.10	0.92 ± 0.10	0.92 ± 0.06
**XCovNet Classifier**				
	**Original Data Only**		**WGAN + Original Data**	
**Accuracy**	58.98% ± 18.77%		93.93% ± 4.06%	
	**Sensitivity**	**Specificity**	**Sensitivity**	**Specificity**
COVID-19	0.46 ± 0.28	0.92 ± 0.07	0.91 ± 0.11	0.97 ± 0.05
Bact. Pneu.	0.46 ± 0.36	0.95 ± 0.07	0.97 ± 0.03	0.97 ± 0.04
Healthy	0.78 ± 0.21	0.87 ± 0.10	0.92 ± 0.10	0.92 ± 0.06

**Table 5:** Results for the classifier model trained using a mix of original and Pix2Pix data for the three input maps used.

**Pretrained VGG16 Classifier**						
	**Canny Edges**		**Sobel Edges**		**Composite Label**	
**Accuracy**	93.19% ± 3.54%		93.07% ± 3.54%		88.92% ± 5.50%	
	**Sensitivity**	**Specificity**	**Sensitivity**	**Specificity**	**Specificity**	**Sensitivity**
COVID-19	0.94 ± 0.07	0.96 ± 0.04	0.93 ± 0.08	0.97 ± 0.05	0.89 ± 0.11	0.97 ± 0.04
Bact. Pneu.	0.96 ± 0.07	0.97 ± 0.03	0.96 ± 0.06	0.96 ± 0.04	0.90 ± 0.12	0.94 ± 0.04
Healthy	0.90 ± 0.10	0.96 ± 0.04	0.91 ± 0.09	0.97 ± 0.03	0.88 ± 0.11	0.93 ± 0.11
**XCovNet Classifier**						
	**Canny Edges**		**Sobel Edges**		**Composite Label**	
**Accuracy**	91.11% ± 7.11%		91.33% ± 5.36%		85.74% ± 6.12%	
	**Sensitivity**	**Specificity**	**Sensitivity**	**Specificity**	**Specificity**	**Sensitivity**
COVID-19	0.92 ± 0.09	0.91 ± 0.13	0.93 ± 0.05	0.96 ± 0.04	0.87 ± 0.07	0.94 ± 0.04
Bact. Pneu.	0.94 ± 0.04	0.96 ± 0.04	0.89 ± 0.10	0.97 ± 0.03	0.80 ± 0.21	0.96 ± 0.04
Healthy	0.83 ± 0.25	0.96 ± 0.03	0.91 ± 0.10	0.94 ± 0.06	0.88 ± 0.13	0.88 ± 0.09

**Table 6:** Results for the classifier models trained using a mix of original, Pix2Pix, and WGAN data for the three input maps used.

**Pretrained VGG16 Classifier**						
	**WGAN + Canny Edges**		**WGAN + Sobel Edges**		**WGAN + Composite Label**	
**Accuracy**	96.32% ± 4.17%		95.69% ± 3.14%		95.15% ± 3.33%	
	**Sensitivity**	**Specificity**	**Sensitivity**	**Specificity**	**Specificity**	**Sensitivity**
COVID-19	0.95 ± 0.07	0.97 ± 0.07	0.95 ± 0.07	0.95 ± 0.07	0.95 ± 0.08	0.95 ± 0.07
Bact. Pneu.	0.95 ± 0.09	0.99 ± 0.01	0.94 ± 0.8	0.99 ± 0.01	0.93 ± 0.09	0.99 ± 0.02
Healthy	0.97 ± 0.05	0.97 ± 0.03	0.93 ± 0.10	0.97 ± 0.04	0.93 ± 0.09	0.97 ± 0.03
**XCovNet Classifier**						
	**WGAN + Canny Edges**		**WGAN + Sobel Edges**		**WGAN + Composite Label**	
**Accuracy**	93.33% ± 4.92%		93.48% ± 6.97%		94.76% ± 4.12%	
	**Sensitivity**	**Specificity**	**Sensitivity**	**Specificity**	**Specificity**	**Sensitivity**
COVID-19	0.91 ± 0.11	0.98 ± 0.03	0.92 ± 0.08	0.97 ± 0.05	0.92 ± 0.12	0.99 ± 0.03
Bact. Pneu.	0.96 ± 0.04	0.97 ± 0.03	0.97 ± 0.04	0.98 ± 0.03	0.98 ± 0.02	0.97 ± 0.05
Healthy	0.95 ± 0.06	0.94 ± 0.05	0.92 ± 0.10	0.95 ± 0.05	0.92 ± 0.10	0.96 ± 0.07

**Table 7:** Comparison with other studies of LUS classification for COVID-19 screening.

	Accuracy	COVID-19 Sensitivity	COVID-19 Specificity
WGAN + Pix2Pix Canny + VGG16	96.32% ± 4.17%	0.96 ± 0.07	0.97 ± 0.07
WGAN + Pix2Pix Composite + XCovNet	94.76% ± 4.12%	**0.99 ± 0.03**	0.92 ± 0.12
Born et al. [19]—Images	87.80%	0.88 ± 0.07	0.94 ± 0.05
Born et al. [19]—Videos	90.00%	0.90 ± 0.08	0.96 ± 0.04
Awasthi et al. [20]	83.20%	0.92	0.68
Barros et al. [17]	93.13%	0.97 ± 0.06	0.96 ± 0.09
Karar et al. [54]	99.45%	**0.99**	**1.00**
Bruno et al. [77]	**100.00%**	-	-
Zhang et al. [56]	91.00% ± 3.00%	0.88 ± 0.07	0.90 ± 0.08
Madhu et al. [21]	99.76%	**0.99**	0.99
Zeng et al. [22]	90.60%	0.83	-

Highest values for each measure are bolded.

**Table 8:** CAMs centroids’ average and dispersion.

		(${\bar{c}}_{x},{\bar{c}}_{y}$)	σx	σy
	COVID-19	(59.21, 45.27)	11.97	13.49
VGG16 (Original Data Only)	Bact. Pneu.	(57.33, 51.86)	7.65	11.17
	Regular	(55.82, 54.03)	10.70	15.12
	COVID-19	(53.47, 43.15)	13.74	19.51
XCovNet (Original Data Only)	Bact. Pneu.	(55.63, 55.27)	12.77	15.91
	Regular	(50.19, 63.78)	8.70	9.77
	COVID-19	(60.16, 51.11)	13.09	16.05
WGAN + Pix2Pix Canny + VGG16	Bact. Pneu.	(56.26, 50.30)	8.61	10.84
	Regular	(56.43, 62.65)	8.82	15.34
	COVID-19	(53.91, 47.96)	14.16	19.03
WGAN + Pix2Pix Composite + XCovNet	Bact. Pneu.	(58.29, 51.25)	7.96	10.55
	Regular	(53.16, 58.27)	13.58	18.20

## Data Availability

The data presented in this study are available in Born et al. [19], and can be openly accessed in https://github.com/jannisborn/covid19_ultrasound (accessed on 22 November 2025). The code developed can be accessed in https://github.com/pedrosergiot/gan_covid_repo (accessed on 22 November 2025).

## Abbreviations

The following abbreviations are used in this manuscript:

GAN	Generative adversarial network
WGAN	Wasserstein GAN
SARS-CoV-2	Severe acute respiratory syndrome coronavirus 2
WHO	World Health Organization
COVID-19	Coronavirus disease 2019
RT-PCR	Reverse transcription polymerase chain reaction
CT	Computerized tomography
AI	Artificial intelligence
LUS	Lung ultrasound
SVM	Support vector machine
ROC	Receiver operating characteristic
CNN	Convolutional neural network
LSTM	Long short-term memory
ProGAN	Progressive GAN
SRGAN	Super-resolution GAN
RFI-GAN	Reference-guided Fuzzy integral GAN
SA-GAN	Supervised autoencoder GAN
MS-SSIM	Multiscale structural similarity
POCUS	Point-of-care ultrasound
ANN	artificial neural network
WGAN-GP	Wasserstein GAN with gradient penalty
ReLU	Rectified linear
cGAN	Conditional GAN
Grad-CAM	Gradient-weighted Class Activation Mapping
KL	Kullback–Leiber
KID	Kernel inception distance
FID	Fréchet inception distance
